# Reproducibility and responsiveness of the Frailty Index and Frailty Phenotype in older hospitalized patients

**DOI:** 10.1186/s12877-021-02444-y

**Published:** 2021-09-17

**Authors:** Marlies Feenstra, Frederike M.M. Oud, Carolien J. Jansen, Nynke Smidt, Barbara C. van Munster, Sophia E. de Rooij

**Affiliations:** 1 Department of Internal Medicine and Geriatrics, University of Groningen, University Medical Center Groningen, PO Box 30001, HPC: AA43, 9700 RB Groningen, The Netherlands; 2 Medical Spectrum Twente Hospital, Enschede, the Netherlands; 3 Department of Geriatrics, Gelre Hospitals, Apeldoorn, The Netherlands; 4 Department of Epidemiology, University of Groningen, University Medical Center Groningen, Groningen, The Netherlands

**Keywords:** Frail, Psychometric properties, Measurement properties, Reliability, Internal Medicine, Geriatric care

## Abstract

**Background:**

There is growing interest for interventions aiming at preventing frailty progression or even to reverse frailty in older people, yet it is still unclear which frailty instrument is most appropriate for measuring change scores over time to determine the effectiveness of interventions. The aim of this prospective cohort study was to determine reproducibility and responsiveness properties of the Frailty Index (FI) and Frailty Phenotype (FP) in acutely hospitalized medical patients aged 70 years and older.

**Methods:**

Reproducibility was assessed by Intra-Class Correlation Coefficients (ICC), standard error of measurement (SEM) and smallest detectable change (SDC); Responsiveness was assessed by the standardized response mean (SRM), and area under the receiver operating characteristic curve (AUC).

**Results:**

At baseline, 243 patients were included with a median age of 76 years (range 70–98). The analytic samples included 192 and 187 patients in the three and twelve months follow-up analyses, respectively. ICC of the FI were 0.85 (95 % confidence interval [CI]: 0.76; 0.91) and 0.84 (95% CI: 0.77; 0.90), and 0.65 (95% CI: 0.49; 0.77) and 0.77 (95% CI: 0.65; 0.84) for the FP. SEM ranged from 5 to 13 %; SDC from 13 to 37 %. SRMs were good in patients with unchanged frailty status (< 0.50), and doubtful to good for deteriorated and improved patients (0.43–1.00). AUC’s over three months were 0.77 (95% CI: 0.69; 0.86) and 0.71 (95% CI: 0.62; 0.79) for the FI, and 0.68 (95% CI: 0.58; 0.77) and 0.65 (95% CI: 0.55; 0.74) for the FP. Over twelve months, AUCs were 0.78 (95% CI: 0.69; 0.87) and 0.82 (95% CI: 0.73; 0.90) for the FI, and 0.78 (95% CI: 0.69; 0.87) and 0.75 (95% CI: 0.67; 0.84) for the FP.

**Conclusions:**

The Frailty Index showed better reproducibility and responsiveness properties compared to the Frailty Phenotype among acutely hospitalized older patients.

**Supplementary Information:**

The online version contains supplementary material available at 10.1186/s12877-021-02444-y.

## Background

Frailty is a medical condition of increased vulnerability and poor resolution of homeostasis after a stressor event as a consequence of cumulative decline in many physiological systems during a lifetime [1]. Around 40 % of the hospitalized older patients are frail which is associated with poor health outcomes, such as functional decline, hospital re-admission, institutionalization, and mortality [2, 3].

Identifying (pre) frail older adults, and those at risk for progression of frailty is important. Some older adults may benefit from interventions targeted at prevention of frailty progression to lower the risk of poor health outcomes like functional decline [4, 5]. Reliable and valid assessment of frailty and how to measure relevant changes in frailty over time is therefore crucial.

Several frailty instruments exist for the purpose of diagnosing, risk stratification, and evaluating frailty over time [6]. Comprehensive geriatric assessment is currently the gold standard for diagnosing the frailty status in clinical practice [1], but the cumulative deficits model or Frailty Index (FI) and the Frailty Phenotype (FP) are the most widely used instruments used to establish frailty status in research [7, 8]. Construct validity and predictive validity of negative health outcomes of the FI and FP have been extensively evaluated and are proven to be satisfactory in both community-dwelling and hospitalized older adults [9–11]. Reproducibility and responsiveness of change scores of frailty instruments are poorly studied especially after hospitalization and it is still unclear which frailty instrument is most appropriate for measuring change scores over time or the effectiveness of interventions [9, 11, 12].

Therefore, the aim of this study is to determine the reproducibility and responsiveness of the FI and FP in acutely admitted hospitalized older medical patients.

## Methods

### Study population

During this monocentric prospective cohort study, patients aged ≥ 70 years were recruited between February 2017 and May 2018. Twelve months’ follow-up measurements continued to the end of April 2019. During weekdays patients admitted to surgical, cardiology, pulmonology, medical oncology, nephrology, and general internal medicine wards were checked for eligibility to participate. Inclusion criteria were age ≥ 70 years and an expected hospital stay of at least two days. Exclusion criteria were no understanding of the Dutch language, any (temporary) cognitive condition that influenced decision making capacity, and no written informed consent. The Research Ethics Committee of the University Medical Center Groningen ruled that no formal ethics approval was required (file number: 201,600,268). All participants provided written informed consent before participation.

### Data collection

Baseline assessment took place within four days after admission. Telephonic assessments were performed at three and twelve months’ post-discharge, in which baseline questions were repeated and two anchor questions were added [13]. Data were collected by trained research staff.

### Questionnaires

#### Frailty instruments

The FI score was calculated using 34 deficits associated with health status [14]. The FP was assessed by five self-reporting criteria including strength, walking ability, weight loss, physical activity, and exhaustion [15, 16]. A detailed description of the included items of the FI and FP are presented in Additional File 1, Tables A1 and A2.

#### Patient-reported anchor questions

Anchor questions were used as an external criterion for measuring responsiveness [17]. Two different anchor questions were used: (1) ‘In general, how is your health state now, compared to three months/twelve months ago before hospitalization?’ (2) ‘In general how is your daily functioning now, compared to three months/twelve months ago before hospitalization?’ Response options were a five point Likert scale. Patients were divided into three categories based on the anchor questions (improved, unchanged, and deteriorated patients). Improved was scored if a patient answered ‘slightly better’ or ‘much better’. Unchanged was scored if the answer was ‘more or less the same’. Deteriorated was scored if a patient answered ‘slightly worse’ or ‘much worse’.

#### Sociodemographic and patient characteristics

For all subjects, baseline sociodemographic characteristics were collected including age, sex, living situation (independent living vs. not independent living), and educational level (≤ high school vs. > high school. After discharge, medical charts were consulted to assess baseline comorbidity (Charlson Comorbidity Index) [18] and mortality during follow-up time.

### Statistical analysis

#### Descriptive statistics

For all baseline sociodemographic and patient characteristics, descriptive statistics were calculated. The distribution of the scores on frailty instruments at baseline were inspected for possible floor and ceiling effects. Thresholds for floor and ceiling effects were if ≥ 15 % of the patients achieved the lowest or highest possible score, respectively [19].

#### Reproducibility

Test-retest reproducibility was assessed among patients who reported to be unchanged according to the anchor question three months post discharge. The following parameters were calculated:

- The intraclass correlation coefficient (ICC) using a two-way mixed effects model for absolute agreement was used for the baseline and three months follow-up measurements of the FI and the FP. Cut-off values for interpretation of the ICC including the 95 % confidence interval were < 0.5 poor, ≥ 0.5 and < 0.75 moderate, ≥ 0.75 and < 0.9 good, ≥ 0.9 excellent reliability [20].
- Cohen’s kappa and absolute agreement were calculated to assess the reproducibility of the FP using the categorized outcome (robust, prefrail, frail). Cut-off values were < 0.40 poor, ≥ 0.40 and < 0.75 fair to good, ≥ 0.75 excellent agreement [19].
- Measurement error of the FI and FP was assessed by calculating Bland-Altman plots and the standard error of measurement (SEM). Bland-Altman plots were calculated by the mean change scores of baseline and three months post discharge assessments plotted against the difference on both scores. SEM was calculated using the following formula: SEM = SD(T0) x √(1-r). SD is the standard deviation of the baseline measurement of the Frailty Index of the unchanged group; r refers to the ICC. To interpret the SEM, scores are converted to percentages of the scale range. Cut-off values were: ≤5 % very good, ≤ 10 % good, > 10 % and < 20 % doubtful, ≥ 20 % poor [21].
- To be able to interpret change scores, the smallest detectable change (SDC) was calculated for the FI and FP using continuous scores. SDC reflects the variance of the distribution of change scores among stable patients. Patients who reported no change according to the anchor question were assumed to be stable. SDC was calculated by the following formula: SDC = 1.96 x √2 x SEM [21]. Both the absolute SDC value as well as the SDC as a percentage of the scale range were calculated.

#### Responsiveness

Two types of responsiveness were determined over the timeframes from pre-hospital admission to three and twelve months’ post discharge:

1. Internal responsiveness, defined as the magnitude of change related to the variance in change scores, was determined by the standardized response mean (SRM). SRM is calculated by dividing the mean change score by the standard deviation of the mean change score [22]. SRMs were separately calculated for improved, unchanged, and deteriorated patients according to the anchor question for both the FI and FP using continuous scores. Cut-off values were: ≤0.2 small, > 0.2 and ≤ 0.5 doubtful, > 0.5 and ≤ 0.8 good, > 0.8 very good internal responsiveness for the improved and deteriorated patients; <0.50 good, ≥ 0.50 small for the unchanged patients [22].
2. External responsiveness, defined as the ability to detect change over time in the construct to be measured, was assessed by investigating the ability of the instruments to discriminate between relevant changes (improved and deteriorated patients) and irrelevant changes (unchanged patients) [19]. This is reflected by the area under the receiver operating characteristic curve (AUC). For these analyses, the anchor question was considered as the gold standard for change, and the change scores on the FI and FP using continuous scores were considered as the ‘diagnostic test’ for measuring change. An AUC of ≥ 0.70 was considered to be adequate. In addition, for each instrument the optimal cut-off point was calculated for which the sensitivity and specificity together revealed the least error in classifying patients as improved versus unchanged and deteriorated versus unchanged. To carry out these analyses, the correlation (Spearman’s rho) between the change score on the frailty instrument and the anchor question should be at least 0.40 [23].

#### Missing data and sensitivity analysis

Patients who died during follow-up were included in the analytic sample by imputing the highest prevalent frailty category for each frailty instrument and the worst outcome for the anchor questions. A complete case analysis was included as a sensitivity analysis. For all analyses IBM SPSS Statistics, version 23 was used.

## Results

### Descriptive statistics

Baseline characteristics were presented in Table 1. Of the 243 participants with baseline assessments, 51 had no follow-up data after three months post discharge resulting in an analytic sample of 192 in the three months follow-up analyses. The analytic sample of the twelve months follow-up analyses included 187 participants. A flowchart including a detailed description of the reason for missing data is provided in Additional File 1, Figure A1. In total, 39 patients (16 %) died during the study. These patients were older and had higher frailty and comorbidity index scores at baseline compared to patients with complete data for all assessments (*n* = 118) and patients lost to follow-up after twelve months (*n* = 56) (Additional File 2, Table S1). Between the latter two groups, no differences in baseline characteristics were found (Additional File 2, Table S1).

**Table 1:** Baseline characteristics of baseline sample, the subsample used for the 3 months post discharge (T1) analyses, and the subsample used for the twelve months post discharge (T2) analyses

**Baseline characteristics**	**Baseline sample**	**T1 sample**	**T2 sample**	
	**(*n* = 243)**	**(*n* = 192)**	**(*n* = 187)**	
**Age, median (IQR 25;75)**	76 (72;81)	76 (72;81)	76 (73;81)	
range (years)	70–98	70–98	70–98	
**Sex, male**	165 (68)	132 (69)	129 (69)	
**Housing situation**				
independent	225 (93)	179 (93)	173 (93)	
not independent	18 (8)	13 (7)	14 (8)	
**Education**				
≤ high school	173 (71)	140 (73)	132 (71)	
> high school	70 (29)	52 (27)	55 (29)	
**CCI, median (IQR 25;75)**	2 (1; 4)	2 (1; 4)	2 (1; 4)	
**Frailty Index**				
median (IQR 25;75)	0.18 (0.08; 0.31)	0.17 (0.08; 0.30)	0.18 (0.08; 0.31)	
lowest possible score	0 (0)	0 (0)	0 (0)	
highest possible score	0 (0)	0 (0)	0 (0)	
**Frailty Phenotype**				
median (IQR 25;75)	1.00 (0; 2.00)	1.00 (0; 2.00)	1.00 (0; 2.00)	
robust (0 criteria)	97 (40)	81 (42)	74 (40)	
prefrail (1 or 2 criteria)	88 (36)	69 (36)	68 (36)	
frail (≥ 3 criteria)	47 (19)	38 (20)	37 (20)	
lowest possible score	97 (40)	81 (42)	74 (40)	
highest possible score	4 (2)	3 (2)	4 (2)	

Notes: Values are presented as numbers and percentage (%) unless indicated otherwise. Percentages may not equal 100 % due to rounding and missing. CCI, Charlson Comorbidity Index; IQR, inter quartile range

The FP showed a floor effect at baseline assessment, hampering detection of improvement in 40 % of these patients (Table 1; Additional File 1, Figure A3). Mean scores of the frailty instruments at baseline and follow-up measurements for all and within collapsed categories as used in the analyses are presented in Table 2 and in Additional File 1, Table A3.

**Table 2:** Mean (SD) frailty scores at baseline (T0) and 3 months post discharge (T1)

	Frailty Index					Frailty Phenotype	
	**n**		**T0** ** mean (SD)**	**T1** **mean (SD)**		**T0** **mean (SD)**	**T1** **mean (SD)**
**Health anchor**							
**All categories**							
much better	24		0.19 (0.12)	0.13 (0.09)		1.38 (1.28)	0.35 (0.65)
slightly better	24		0.23 (0.19)	0.22 (0.20)		1.57 (1.27)	1.00 (1.18)
more or less the same	74		0.16 (0.12)	0.13 (0.11)		0.73 (1.12)	0.67 (0.98)
slightly worse	34		0.19 (0.15)	0.22 (0.16)		0.97 (1.36)	1.09 (1.36)
much worse	36		0.32 (0.17)	0.60 (0.25)		2.14 (1.40)	3.60 (1.79)
**Collapsed categories**^**a.**^							
improved	48		0.21 (0.16)	0.17 (0.16)		1.48 (1.27)	0.68 (1.00)
unchanged	74		0.16 (0.12)	0.13 (0.11)		0.73 (1.12)	0.67 (0.98)
deteriorated	70		0.26 (0.17)	0.42 (0.28)		1.57 (1.49)	2.38 (2.02)
**Functioning anchor**							
**All categories**							
much better	16		0.19 (0.13)	0.13 (0.09)		1.60 (1.30)	0.25 (0.45)
slightly better	21		0.19 (0.18)	0.15 (0.17)		1.29 (1.42)	0.70 (1.03)
more or less the same	80		0.17 (0.14)	0.16 (0.14)		0.78 (1.15)	0.74 (0.99)
slightly worse	40		0.21 (0.15)	0.22 (0.16)		1.21 (1.32)	1.08 (1.37)
much worse	35		0.32 (0.15)	0.61 (0.23)		2.12 (1.39)	3.82 (1.57)
**Collapsed categories**^**a.**^							
improved	37		0.19 (0.16)	0.14 (0.14)		1.42 (1.36)	0.50 (0.84)
unchanged	80		0.17 (0.14)	0.16 (0.14)		0.78 (1.15)	0.74 (0.99)
deteriorated	75		0.26 (0.16)	0.40 (0.28)		1.64 (1.42)	2.34 (2.00)

^a^The collapsed categories were used in the analyses of the health state and functioning anchor questions

### Reproducibility

ICCs, SEMs, and SDCs were presented in Table 3. ICC of the FI were 0.85 (95 % confidence interval [CI]: 0.76; 0.91) and 0.84 (95% CI: 0.77; 0.90), and 0.65 (95% CI: 0.49; 0.77) and 0.77 (95% CI: 0.65; 0.84) for the FP. Kappa statistics of the FP categories were 0.41 (absolute agreement: 0.68) and 0.45 (absolute agreement: 0.70) indicating fair agreement (Additional File 1, Tables A4 – A7). SEM of the FI were good to very good (5 and 6 %) and doubtful for the FP (11 and 13 %) (Table 3). Bland Altman plots are presented in the Additional File 1, Figures A4 and A5. Good agreement was observed for the FI using the functioning anchor and for the FP using the health and functioning anchors (*p* > 0.05). A systematic mean difference was observed between baseline and three-months follow-up tests of the FI using the health anchor (mean difference 0.02; 95 % CI: 0.01; 0.03).

**Table 3:** Reproducibility properties of unchanged patients at three months follow-up measurement

**Health anchor (*n* = 74)**					
**Instrument**	**ICC (95 % CI)**	**SEM**	**SEM%**^**a**^	**SDC**	**SDC%**^**a**^
**FI**	0.85 (0.76; 0.91)	0.05	5 %	0.13	13 %
**FP**	0.65 (0.49; 0.77)	0.66	13 %	1.84	37 %
**Functioning anchor (*n* = 80)**					
**Instrument**	**ICC (95 % CI)**	**SEM**	**SEM%**^**a**^	**SDC**	**SDC%**^**a**^
**FI**	0.84 (0.77; 0.90)	0.06	6 %	0.16	16 %
**FP**	0.77 (0.65; 0.84)	0.56	11 %	1.56	31 %

Notes: Intraclass correlation coefficient for agreement using a 2 way mixed effect model. *ICC* Intraclass Correlation Coefficient, *FI* Frailty Index; *FP* Frailty Phenotype; *SDC* smallest detectable change; *SEM* standard error of measurement.

^a^. SEM% and SDC% are SEM and SDC expressed in percentages of the continuous score of the instrument

### Responsiveness

All SRMs in the improved and deteriorated groups were higher than the SRMs in the stable (unchanged) groups, meaning that the measured change in frailty outcomes was lower among patients in the stable groups compared to patients in both the improved and deteriorated groups (Table 4). Largest SRMs were found for deterioration over twelve months (Table 4), with SRMs ranging from 0.69 to 1.00, indicating good to very good internal responsiveness.

**Table 4:** Mean change scores and internal responsiveness for improved, unchanged, and deteriorated patients

**Health anchor**	**3 months post discharge (*n* = 192)**			**12 months post discharge (*n* = 187)**		
	**mean change score (SD)**	**Correlation**	**SRM**	**Mean change score (SD)**	**Correlation**	**SRM**
**Frailty Index**						
improved	-0.05 (0.11)	0.48	-0.45	-0.07 (0.12)	0.61	-0.58
unchanged	-0.02 (0.06)		-0.33	-0.01 (0.08)		-0.13
deteriorated	0.16 (0.23)		0.70	0.27 (0.27)		1.00
**Frailty Phenotype**						
improved	-0.86 (1.21)	0.46	-0.71	-0.93 (1.38)	0.57	-0.67
unchanged	-0.05 (0.88)		-0.06	-0.18 (0.86)		-0.21
deteriorated	0.82 (1.67)		0.49	1.60 (2.00)		0.80
**Functioning anchor**	**3 months post discharge (*n* = 192)**			**12 months post discharge (*n* = 187)**		
	**mean change score (SD)**	**Correlation**	**SRM**	**Mean change score (SD)**	**Correlation**	**SRM**
**Frailty Index**						
improved	-0.06 (0.11)	0.43	-0.55	-0.08 (0.13)	0.57	-0.62
unchanged	-0.01 (0.06)		-0.16	-0.01 (0.09)		-0.11
deteriorated	0.13 (0.23)		0.57	0.23 (0.27)		0.85
**Frailty Phenotype**						
improved	-0.94 (1.31)	0.47	-0.72	-1.17 (1.46)	0.57	-0.80
unchanged	-0.09 (0.73)		-0.12	-0.24 (1.01)		-0.24
deteriorated	0.74 (1.73)		0.43	1.36 (1.96)		0.69

Notes: Spearman correlation coefficients were calculated by using the mean change scores of the total group and the anchor question. Abbreviations: *SD* standard deviation; *SRM* standardized response mean

**Fig. 1:**
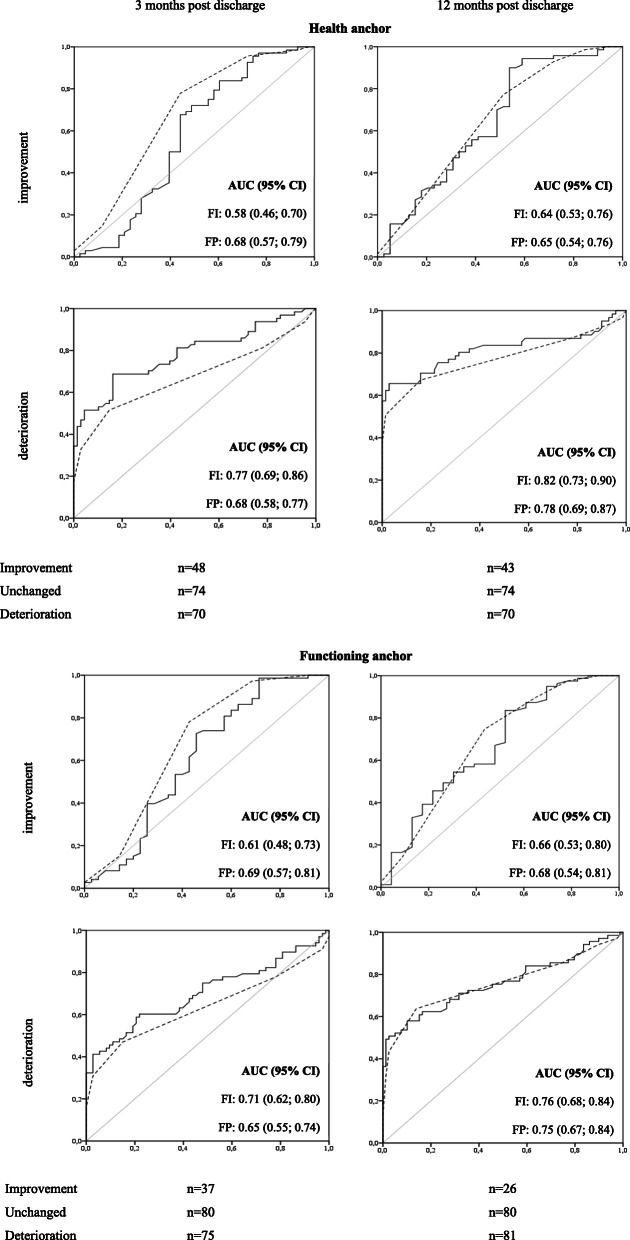
Receiver Operating Characteristic (ROC) curve comparisons and corresponding Area Under the ROC curves (AUC) between the Frailty Index (solid line) and the Frailty Phenotype (dashed line) for measuring improvement and deterioration in frailty status according to the health and functioning anchor questions after three (left) and twelve (right) months post discharge. The diagonal lines (gray) represent the reference lines of no-discrimination

Only the FI showed sufficient responsiveness (> 0.70) to detect deterioration in frailty over three months (Fig. 1). Highest AUC of the FI was found for the health anchor question (AUC: 0.77; 95 % CI: 0.69; 0.86, see Fig. 1). The optimal cut-off of the change scores from baseline to three months post discharge was 0.02 with corresponding sensitivity and specificity of 69 and 81 % (Additional File 1, Table A8). Over the timeframe of twelve month, both the FI and FP were responsive for deterioration in frailty status. Highest AUC’s were found for the health anchor (AUC: 0.82; 95 % CI: 0.73; 0.90 and AUC: 0.78; 95 % CI: 0.69; 0.87) (Fig. 1) with corresponding optimal change score of 0.04 (Sensitivity: 75 %; Specificity: 77 %) and 0.50 (Sensitivity: 68 %; Specificity: 84 %) for the FI and FP, respectively (Additional File 1, Table A8). The FP consistently performed better in detecting improvement in frailty, yet all AUC values were below the threshold of 0.70 (Fig. 1).

Sensitivity analyses including complete cases only yielded essentially the same results. However, as expected, by disregarding the frailest individuals (Additional File 2, Table S1) and due to the smaller sample, the AUC values regarding deterioration were lower in this subgroup (Additional File 2, Figure S1).

## Discussion

In this study on acutely hospitalized older medical patients, we found that the FI showed good reproducibility and the FP showed moderate reproducibility. In addition, the FI was responsive in detecting deterioration in frailty between pre-acute hospital admission and three and twelve months post discharge. The FP was only responsive in detecting patients with deteriorating frailty status between pre-hospital admission and twelve months post-discharge.

Like cholesterol is a measurable marker for cardiovascular disease risk and HbA1c for risk of complications of diabetes type 2, frailty can be considered as the measurable marker for risk of adverse outcomes. Responsive frailty measures can therefore act as an important intermediate outcome for the evaluation of interventions aiming at preventing adverse health outcomes in older patients. Despite that the FP is frequently used as an outcome instrument to evaluate interventions, we failed to establish satisfactory reproducibility and responsiveness of the FP over a three months timeframe [24–26]. In addition, a floor effect of the FP was found in 40 % of the cases. This may have led to an underestimation of the ability to detect improvements, but it may have overestimated the results based on the unchanged patients. Consequently, the reproducibility outcomes may have been even overestimated due to the floor effect of the FP. Although, in theory, an instrument can be responsive with a low validity or reliability, it may be undesirable to use such an instrument to measure change over time [27]. Consequently, despite the apparently better outcomes of the FP compared to the FI to measure improvements in frailty status over time, we have substantial reservations to draw this conclusions due to the unsatisfactory reproducibility outcomes of the FP in the current study. In addition, the practical applicability of the FP to measure improvement over time is limited by the presence of a floor effect of the FP.

In the current study, an increase of five deficits over three months and six deficits over twelve months corresponds with the smallest detectable change of the FI that is meaningful to patients. The found change scores are higher than reported in recent other studies investigating clinically meaningful change of the FI [28, 29]. These differences may be explained by the used anchor: in the current study a subjective perception of change in health and functioning according to the patient was used, whereas the other studies used existing instruments, like the EQ-5D and the Clinical Frailty Scale, as the external anchor. Our participants could have been adapted to their changed health state after hospitalization [30] which may have resulted in a larger change score that is regarded meaningful to patients themselves. However, patients who perceived no change in health and functional status after three and twelve months post discharge, also had the smallest change scores on the frailty instruments. Another explanation may be that the change in frailty status according to a clinician which was investigated by Theou and colleagues [29] is different from the patients perception of change in frailty status as investigated in the current study.

Strengths of this study are the use of various methods for assessing reproducibility and responsiveness and the large sample size. More than twice the recommended number of patients for evaluating psychometric properties were included [19]. There are, however, limitations. First, instead of using a comprehensive geriatric assessment, the gold standard for frailty status, we used two relevant patient-based anchor questions to determine change in frailty. By using two anchor questions referring to change in function and health in a population that is prone to frailty, we intended to come close to evaluating real frailty status. The moderate positive correlations of these anchor questions with the change scores on the frailty instruments indicate that the chosen anchor questions may not fully capture the whole concept of frailty. Directly asking for change in frailty was, however, not an option because older adults themselves are not familiar with this concept [31]. Second, the design for determining the reproducibility of the frailty instruments was not ideal. On the one hand, the time interval between the test and the retest, three months, is a long period to assume that the patients have remained stable. In addition, stability was based on self-reported, thus subjective, anchor questions, which did show a moderate correlation with the change scores on the frailty instruments (ranging from 0.43 to 0.48). Although mean change scores and standard deviations were smallest in the unchanged subgroups, suggesting little variation in frailty scores between the first and second assessments and suggesting clinically stable patients, the gold standard for measuring frailty was unavailable and objective clinical stability could not be guaranteed. However, if some unstable patients were inadvertently considered stable in the current study, the found reproducibility scores are expected to be an underestimation of the “real” reliability. Third, we cannot rule out that the results are biased by response shift, as response shift evaluation was not incorporated in the study design [30]. Fourth, the modified FP was used instead of the original performance-based measures because in our population of older medical patients, performance-based assessment is often too challenging and would have led to the inclusion of the fittest frail patients, resulting in an undesirable selection bias. Fifth, due to ethical considerations, no patients were included with cognitive impairment due to dementia or delirium, although frailty in these patients is often present and their frailty status is expected to decline after hospitalization. However, the broad inclusion criteria still resulted in the inclusion of a representative sample of the geriatric population consisting of a heterogeneous group of mentally competent older medical patients.

Future research should compare the responsiveness of existing frailty instruments and their relation to the course of functional impairment in multiple studies and other patient groups with a reliable gold standard for the measurement of frailty. These studies should also incorporate measures such as a then-test in study designs to identify and adjust for response shift.

## Conclusions

In this study, the Frailty Index showed better reproducibility and responsiveness properties compared to the Frailty Phenotype. Based on this single study we cannot yet formulate concrete recommendations about the best instrument to evaluating frailty status over time that is meaningful to older patients.

## Supplementary Information

**Additional file 1: Table A1.** List of items Frailty Index.** Table A2.** List of the criteria and items Frailty Phenotype. **Figure A1.** Flowchart of subjects. **Figure A2.** Baseline distribution and frequency of Frailty Index scores. **Figure A3.** Baseline distribution and frequency Frailty Phenotype scores. **Table A3.** Mean scores for all and collapsed categories at 12 months follow-up. **Figure A4.** Bland Altman plots health anchor. **Figure A5.** Bland Altman plots functioning anchor. **Table A4.** Cross table Frailty Phenotype and the health anchor. **Table A5.** Kappa statistic Frailty Phenotype health anchor. **Table A6.** Cross tables Frailty Phenotype and functioning anchor. **Table A7.** Kappa statistic Frailty Phenotype functioning anchor. **Table A8.** External responsiveness, AUC, sensitivity and specificity at 12 months follow-up.

**Additional file 2:** Sensitivity analysis including. **Table S1.** Complete case analysis: Baseline characteristics. **Table S2.** Complete case analysis: Reproducibility properties of unchanged patients at three months follow-up measurement. **Table S3.** Complete case analysis: Mean change scores and internal responsiveness for improved, unchanged, and deteriorated patients. **Figure S1.** Complete Case Analysis: Receiver Operating Characteristic (ROC) curve comparisons and corresponding Area Under the ROC curves (AUC).

## Data Availability

The datasets used during the current study are available from the corresponding author upon reasonable request.

## Abbreviations

AUC: Area Under the receiver operating Curve
CCI: Charlson Comorbidity Index
CI: Confidence Interval
ICC: Intra-class Correlation Coefficient
IQR: Inter Quartile Range
FI: Frailty Index
FP: Frailty Phenotype
SD: Standard Deviation
SDC: Smallest Detectable Change
SEM: Standard Error of Measurement
SRM: Standardized Response Mean
