# Supplementary material for: Reproducibility and responsiveness of the Frailty Index and Frailty Phenotype in older hospitalized patients
Source: BMC Geriatr. 2021 Sep 17;21:499. doi: 10.1186/s12877-021-02444-y (PMC8447764; doi:10.1186/s12877-021-02444-y)
Supplement: Supplementary file 1 — Additional file 1: Table A1. List of items Frailty Index. Table A2. List of the criteria and items Frailty Phenotype. Figure A1. Flowchart of subjects. Figure A2. Baseline distribution and frequency of Frailty Index scores. Figure A3. Baseline distribution and frequency Frailty Phenotype scores. Table A3. Mean scores for all and collapsed categories at 12 months follow-up. Figure A4. Bland Altman plots health anchor. Figure A5. Bland Altman plots functioning anchor. Table A4. Cross table Frailty Phenotype and the health anchor. Table A5. Kappa statistic Frailty Phenotype health anchor. Table A6. Cross tables Frailty Phenotype and functioning anchor. Table A7. Kappa statistic Frailty Phenotype functioning anchor. Table A8. External responsiveness, AUC, sensitivity and specificity at 12 months follow-up. [file 12877_2021_2444_MOESM1_ESM.docx]

**ADDITIONAL FILE 1 – SUPPLEMENTARY MATERIAL**

| Table A1: List of items Frailty Index………………………………………………………….. | p. 2 |
| --- | --- |
| Table A2: List of the criteria and items Frailty Phenotype…………………………………….. | p. 5 |
| Figure A1: Flowchart of subjects………………………………………………………………. | p. 6 |
| Figure A2: Baseline distribution and frequency of Frailty Index scores...…………………….. | p. 7 |
| Figure A3: Baseline distribution and frequency Frailty Phenotype scores…………………….. | p. 8 |
| Table A3: Mean scores for all and collapsed categories at 12 months follow-up.………….... | p. 9 |
| Figure A4: Bland Altman plots health anchor.……………………………………………….. | p. 10 |
| Figure A5: Bland Altman plots functioning anchor……………………………………………. | p. 11 |
| Table A4: Cross table Frailty Phenotype and the health anchor……………………………… | p. 12 |
| Table A5: Kappa statistic Frailty Phenotype health anchor……………………………………. | p. 12 |
| Table A6: Cross tables Frailty Phenotype and functioning anchor……………………………. | p. 13 |
| Table A7: Kappa statistic Frailty Phenotype functioning anchor……………………………… | p. 13 |
| Table A8: External responsiveness, AUC, sensitivity and specificity at 12 months follow-up.. | p. 14 |
|  |  |

## **Table A1**: List of 34 items used for the Frailty Index

Deficits were rescaled into values between 0 and 1, representing the presence of the deficits measured. The total score represents present deficits as a proportion of all deficits measured, resulting in a score ranging from 0 to 1.

| **Variable** | **Scoring** |
| --- | --- |
| **ADL / iADL domain** | |
| 1. Help bathing | Yes = 1  No = 0 |
| 1. Help dressing |  |
| 1. Help grooming |  |
| 1. Help using toilet |  |
| 1. Difficulty getting out chair |  |
| 1. Help getting out chair |  |
| 1. Help walking |  |
| 1. Help eating |  |
| 1. Help using phone |  |
| 1. Help transport |  |
| 1. Help shopping |  |
| 1. Help meal preparations |  |
| 1. Help housework |  |
| 1. Help taking medications |  |
| 1. Help finances |  |
| **Geriatric syndrome** | |
| 1. Urinary incontinence | Yes = 1  No = 0 |
| 1. Visual impairment |  |
| 1. Hearing impairment |  |
| 1. Fall in past month |  |
| 1. Unintentional weight loss |  |
| **Psychosocial domain** | |
| 1. Feel everything is an effort | Yes = 1 No = 0 |
| 1. Difficulty get going |  |
| 1. Self-rated health | Poor = 1 Fair = 0.75  Good = 0.5 Very Good = 0.25 Excellent = 0 |
| 1. Did you feel downhearted and blue? 2. Have you dropped many of your interests and activities? | Both yes = 1  One yes = 0.5  Both no = 0 |
| **Symptoms** | |
| 1. Pain / discomfort | No pain / discomfort = 0 Moderate = 0.5 Extreme = 1 |
| 1. Mood | Not anxious / depressed = 0  Moderately = 0.5 Extreme = 1 |
| 1. Loss of appetite^*^ | Yes = 1 No = 0 |
| **Physical domain** | |
| 1. Housing situation | Independent / conventional = 0 Transitional = 0.5  Institutionalized = 1 |
| 1. Living situation | Living alone = 1  Cohabiting/married = 0 |
| 1. Walk outside for 5 minutes | Impossible = 1 Only with help of somebody else = 0.75 Much effort = 0.5 Some effort = 0.25 No effort = 0 |
| 1. Physical exercise for at least 30 minutes | Never = 1 Monthly = 0.75  Weekly = 0.5  3 days/week = 0.25 Daily = 0 |
| 1. Move as intended | Yes = 0  No = 1 |
| **Cognitive domain** | |
| 1. MBT^†^ | Completes test without error <60 sec. = 0  Completes test with error or >60s = 0.33 Engages, but cannot complete test = 0.66 Cannot or will not engage = 1 |

Notes: All baseline assessments referred to the situation two weeks before hospital admission unless indicated otherwise. (i)ADL, (instrumental) Activities of Daily Living; MBT, Months Backwards Test.

* Baseline assessment referred to a month before hospital admission.

^†^ Cognitive functioning was assessed within the first four days of hospital admission.

## **Table A2**: List of the criteria and items used for the Frailty Phenotype.

The sum score of positive criteria were calculated using the international agreed cut-offs: zero criteria indicated robust, one or two criteria indicated prefrail, 3 or more criteria indicated frail phenotype. For some calculations, the FP was considered as a continuous scale (range 0 to 5).

| **Criteria** | **Variable** | **Scoring** |
| --- | --- | --- |
| Weight loss | 1. Report of weight loss of >3 kg in past month | Yes = 1  No = 0 |
| Exhaustion | 1. a. Everything I did was an effort   b. I could not get going | Yes = 1  No = 0  Positive criterion if both are yes |
| Slowness | 1. Not able to walk outside | Yes =1  No = 0 |
| Weakness | 1. Having difficulties rising up a chair | Yes = 1  No = 0 |
| Low physical activity | 1. Being physically active for less than once a week | Yes = 1  No = 0 |

Notes: Baseline assessment referred to the situation two weeks before hospital admission unless indicated otherwise. Cut-offs used: frailty ≥3 positive criteria; prefrail 1 or 2 positive criteria; robust 0 positive criteria.

Baseline n = 243

Analytic sample of the three months post discharge follow-up measurement analyses: n = 192

of which 19 were deceased

Analytic sample of the twelve months post discharge follow-up measurement analyses: n = 187

of which 39 were deceased

Reasons for missing data at first follow-up measurement (n = 30)^b^:

- (Temporary) cognitive or physical problems at moment of FU (n = 6)
- Could not be reached (n = 19)
- Proxy interview (n = 5)

Reasons for missing data at second follow-up measurement (n = 23)^b^:

- (Temporary) cognitive or physical problems at moment of FU (n = 2 )
- Could not be reached (n = 18)
- Proxy interview (n = 3)

Reason for exclusion:

- Withdrawn consent (n = 12)^a^

1. All participants who withdrawn consent during the study have given their explicit consent that the data obtained so far could be used for scientific purposes.
2. Participants with only missing data at the first follow-up measurement (three months discharge) were included in the study sample of the second follow-up measurement (twelve months post discharge).

Reason for exclusion:

- Withdrawn consent (n = 21)^a^

## **Figure A1**: Flowchart and reasons for missing data of subjects.


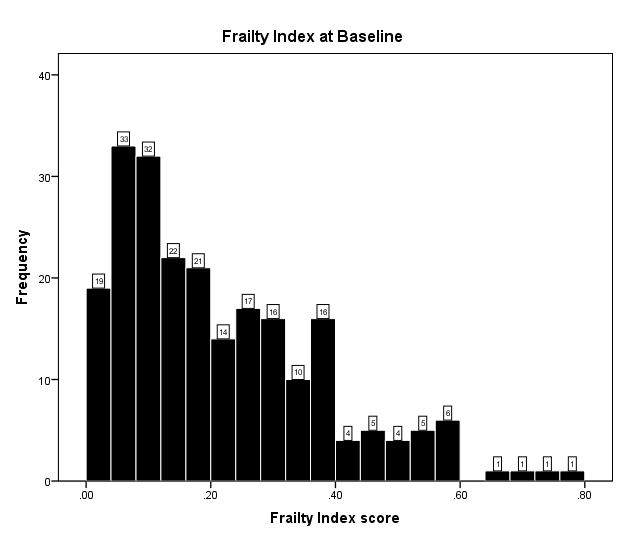


## **Figure A2**: Distribution and frequency of scores of the Frailty Index at baseline (n=228).


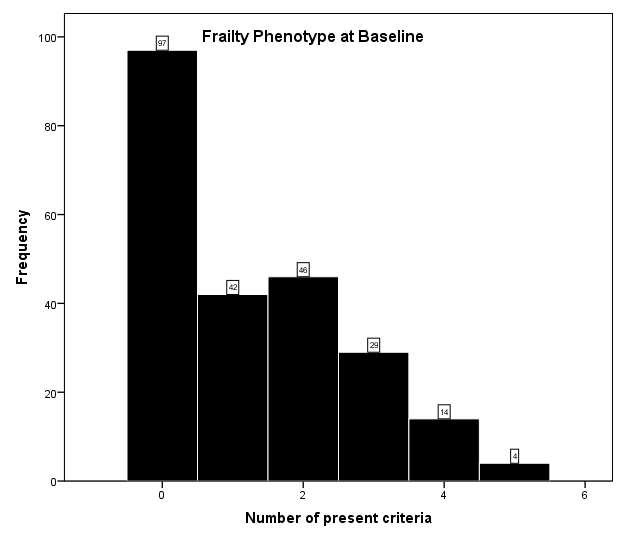


## **Figure A3**: Distribution and frequency of scores of the Frailty Phenotype at baseline (n=232).

| **Table A3:** Mean scores (SD) of the Frailty Index and Frailty Phenotype at baseline (T0) and twelve months post discharge (T2) for all and collapsed categories used in the analyses of the health state and functioning anchor questions. | | | | | | | | | | | |
| --- | --- | --- | --- | --- | --- | --- | --- | --- | --- | --- | --- |
|  | | **Frailty Index** | | | | | |  | **Frailty Phenotype** | | |
|  | | **n** | | **T0  mean (SD)** | **T2 mean (SD)** | |  | | **T0 mean (SD)** | | **T2 mean (SD)** |
| **Health anchor** | | | | | | | | | | | |
| **All categories** | | | | | | | | | | | |
|  | much better | 24 | | 0.15 (0.11) | 0.08 (0.07) | |  | | 1.04 (1.26) | | 0.25 (0.68) |
|  | slightly better | 19 | | 0.22 (0.14) | 0.16 (0.16) | |  | | 1.39 (1.42) | | 0.44 (1.04) |
|  | more or less the  same | 74 | | 0.15 (0.12) | 0.15 (0.12) | |  | | 0.77 (1. 06) | | 0.56 (0.91) |
|  | slightly worse | 21 | | 0.23 (0.16) | 0.24 (0.14) | |  | | 1.20 (1.36) | | 1.40 (1.35) |
|  | much worse | 49 | | 0.32 (0.17) | 0.74 (0.16) | |  | | 2. 36 (1.40) | | 4. 50 (1.26) |
| **Collapsed categories** | |  |  | | |  |  | |  | |  |
|  | improved | 43 | | 0.18 (0.13) | 0.12 (0.13) | |  | | 1.20 (1.33) | | 0.33 (0.85) |
|  | unchanged | 74 | | 0.15 (0.12) | 0.15 (0.12) | |  | | 0.77 (1.06) | | 0.56 (0.91) |
|  | deteriorated | 70 | | 0.31 (0.18) | 0.59 (0.28) | |  | | 2.00 (1.48) | | 3.59 (1.91) |
| **Functioning anchor** | | | | | | | | | | | |
| **All categories** | |  |  | | |  |  | |  | |  |
|  | much better | 15 | | 0.17 (0.11) | 0.06 (0.05) | |  | | 1.36 (1.45) | | 0.07 (0.26) |
|  | slightly better | 11 | | 0.23 (0.18) | 0.17 (0.16) | |  | | 1. 45 (1.37) | | 0.40 (0.70) |
|  | more or less the same | 80 | | 0.15 (0.12) | 0.14 (0.12) | |  | | 0.75 (1.02) | | 0.53 (0.93) |
|  | slightly worse | 25 | | 0.20 (0.17) | 0.18 (0.14) | |  | | 1. 13 (1.42) | | 1.00 (1.31) |
|  | much worse | 56 | | 0.34 (0.17) | 0.68 (0.21) | |  | | 2.21 (1.43) | | 4.16 (1.52) |
| **Collapsed categories** | |  |  | | |  |  | | |  |  |
|  | improved | 26 | | 0.19 (0.14) | 0.10 (0.12) | |  | | 1.40 (1.38) | | 0.20 (0.50) |
|  | unchanged | 80 | | 0.15 (0.12) | 0.14 (0.12) | |  | | 0.75 (1.02) | | 0.53 (0.93) |
|  | deteriorated | 81 | | 0.30 (0.18) | 0.53 (0.30) | |  | | 1.88 (1.51) | | 3.23 (2.06) |

Mean Frailty Index score (Baseline + 3 months FU / 2)

Difference between Frailty Index scores (3 months FU - Baseline)

Mean Frailty Phenotype score (Baseline + 3 months FU / 2)

Difference between Frailty Phenotype scores (3 months FU - Baseline)


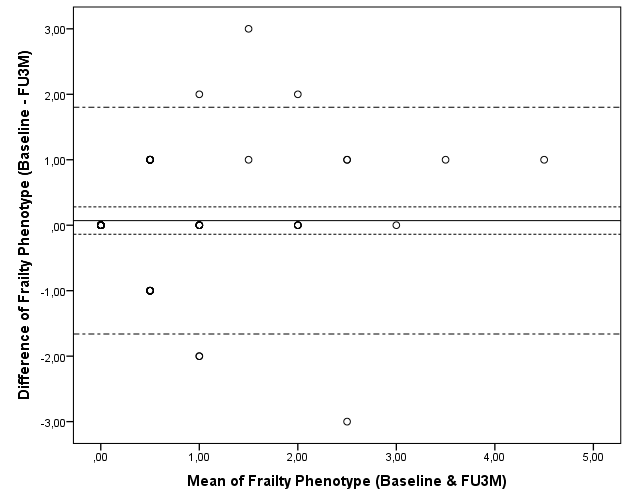

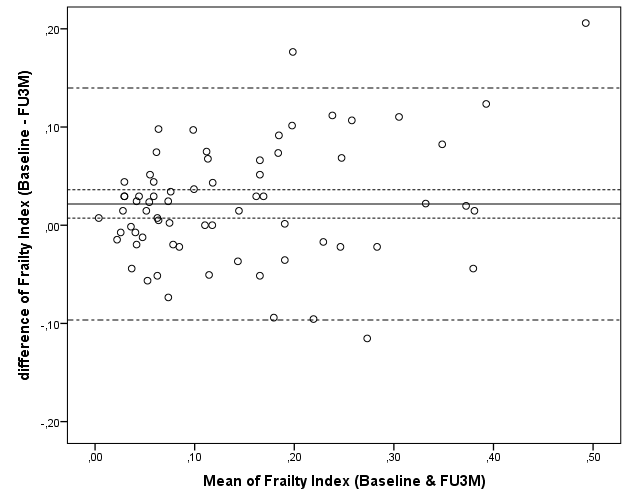


**Figure A4.** Bland Altman plots of the Frailty Index (a) (n=) and Frailty Phenotype (b) for the health anchor in the unchanged group (n=74).

a.

b.


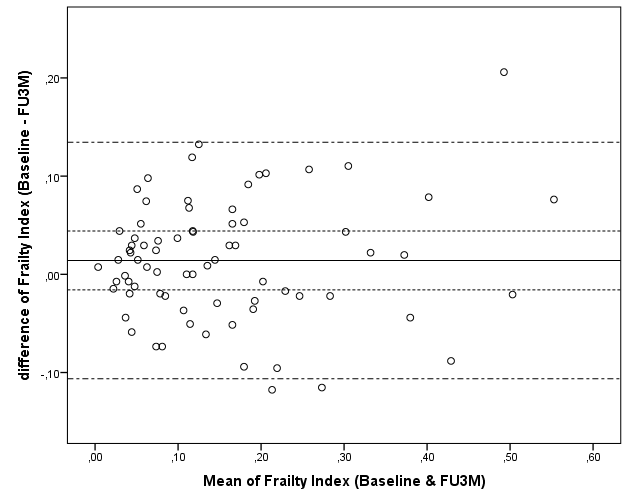


Mean Frailty Index score (Baseline + 3 months FU / 2)

Difference between Frailty Index scores (3 months FU - Baseline)

Mean Frailty Phenotype score (Baseline + 3 months FU / 2)

Difference between Frailty Phenotype scores (3 months FU - Baseline)

**Figure A5.** Bland Altman plots of the Frailty Index (a) and Frailty Phenotype (b) for the functioning anchor in the unchanged group (n=80).

a.

b.


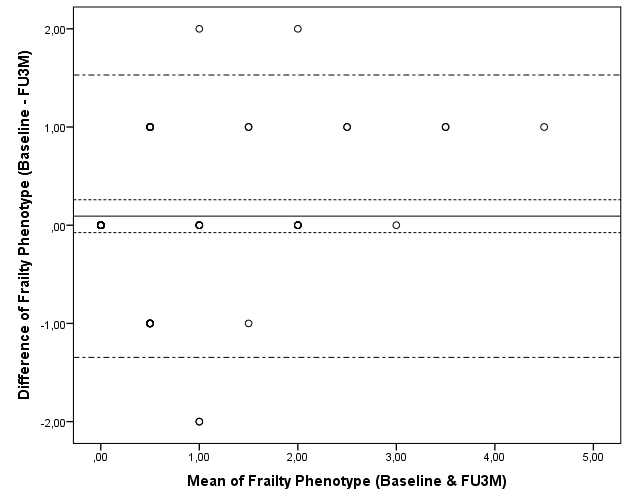


## **Table A4**: Cross table using the categorical scoring of the Frailty Phenotype and the health anchor question in the unchanged group (n=74).

| **Frailty phenotype - Health anchor** | | | **Baseline** | | | **Total** |
| --- | --- | --- | --- | --- | --- | --- |
|  |  |  | **0** | **2** | **3** |  |
| **FU 3 months** | **0** | Count | 32 | 9 | 1 | **42** |
|  |  | Expected Count | 23.8 | 13.6 | 4.5 |  |
|  | **2** | Count | 10 | 14 | 3 | **27** |
|  |  | Expected Count | 15.3 | 8.8 | 2.9 |  |
|  | **3** | Count | 0 | 1 | 4 | **5** |
|  |  | Expected Count | 2.8 | 1.6 | 0.5 |  |
| **Total** | | **Count** | **42** | **24** | **8** | **74** |

Notes: 0 is robust, 2 is prefrail, 3 is frail phenotype. FU, follow-up measurement.

## **Table A5:** Kappa statistic for the categorical scoring of the Frailty Phenotype in unchanged patients according to the health anchor (n=74).

|  | **Value** | **Standard Error^a^** | **p-value** |
| --- | --- | --- | --- |
| **Kappa** | 0.413 | 0.098 | <.001 |

## **Table A6**: Cross tables using the categorical scoring of the Frailty Phenotype and the functioning anchor question in the unchanged group (n=80).

| **Frailty Phenotype - Functioning anchor** | | | **Baseline** | | | **Total** |
| --- | --- | --- | --- | --- | --- | --- |
|  |  |  | **0** | **2** | **3** |  |
| **FU 3 months** | **0** | Count | 35 | 10 | 0 | **45** |
|  |  | Expected Count | 25.9 | 14.6 | 4.5 |  |
|  | **2** | Count | 11 | 16 | 3 | **30** |
|  |  | Expected Count | 17.3 | 9.8 | 3.0 |  |
|  | **3** | Count | 0 | 0 | 5 | **5** |
|  |  | Expected Count | 2.9 | 1.6 | 0.5 |  |
| **Total** | | **Count** | **46** | **26** | **8** | **80** |

Notes: 0 is robust, 2 is prefrail, 3 is frail phenotype. FU, follow-up measurement.

## **Table A7:** Kappa statistic for the categorical scoring of the Frailty Phenotype in unchanged patients according to the functioning anchor (n=80).

|  | **Value** | **Standard Error^a^** | **p-value** |
| --- | --- | --- | --- |
| **Kappa** | .453 | .095 | <.001 |

Notes: Cut-offs are based on highest sensitivity and specificity. Numbers in italic are above the threshold for responsiveness (≥ 0.70). AUC, Area

Under the Curve; FI, Frailty Index; FP, Frailty Phenotype; Se, sensitivity; Sp, specificity.

| **Table A8:** External responsiveness of the Frailty Index and the Frailty Phenotype over three (n=192) and twelve months (n=187) timeframes. | | | | | | | | | |
| --- | --- | --- | --- | --- | --- | --- | --- | --- | --- |
| **Health anchor** | | **3 months post discharge** | | | | **12 months post discharge** | | | |
|  |  | **AUC (95%CI)** | **Cut off** | **Se** | **Sp** | **AUC (95%CI)** | **Cut off** | **Se** | **Sp** |
| **FI** | |  |  |  |  |  |  |  |  |
|  | improvement | 0.58 (0.46; 0.70) | -0.04 | 0.70  0.676  *0.688* | 0.54  0.558  *0.838* | 0.64 (0.53; 0.76) | -0.02 | 0.57 | 0.59 |
|  | deterioration | *0.77 (0.69; 0.86)* | *0.02* | *0.69* | *0.81* | *0.82 (0.73; 0.90)* | *0.04* | *0.75* | *0.77* |
| **FP** | |  |  |  |  |  |  |  |  |
| improvement | | 0.68 (0.57; 0.79) | -0.50 | 0.79 | 0.56 | 0.65 (0.54; 0.76) | -0.50 | 0.77 | 0.49 |
| deterioration | | 0.68 (0.58; 0.77) | 0.50 | 0.52 | 0.84 | *0.78 (0.69; 0.87)* | *0.50* | *0.68* | *0.84* |
| **Functioning anchor** | |  |  |  |  |  |  |  |  |
| **FI** | |  |  |  |  |  |  |  |  |
| improvement | | 0.61 (0.48; 0.73) | -0.03 | 0.62 | 0.56 | 0.66 (0.53; 0.80) | -0.02 | 0.58 | 0.61 |
| deterioration | | *0.71 (0.62; 0.80)* | *0.02* | *0.61* | *0.77* | *0.76 (0.68; 0.84)* | *0.04* | *0.68* | *0.74* |
| **FP** | |  |  |  |  |  |  |  |  |
| improvement | | 0.69 (0.57; 0.81) | -0.5 | 0.78 | 0.59 | 0.68 (0.54; 0.81) | -0.5 | 0.74 | 0.57 |
| deterioration | | 0.65 (0.55; 0.74) | 0.5 | 0.48 | 0.85 | *0.75 (0.67; 0.84)* | *0.5* | *0.63* | *0.86* |
