# Supplementary material for: Reproducibility and responsiveness of the Frailty Index and Frailty Phenotype in older hospitalized patients
Source: BMC Geriatr. 2021 Sep 17;21:499. doi: 10.1186/s12877-021-02444-y (PMC8447764; doi:10.1186/s12877-021-02444-y)
Supplement: Supplementary file 2 — Additional file 2: Sensitivity analysis including. Table S1. Complete case analysis: Baseline characteristics. Table S2. Complete case analysis: Reproducibility properties of unchanged patients at three months follow-up measurement. Table S3. Complete case analysis: Mean change scores and internal responsiveness for improved, unchanged, and deteriorated patients. Figure S1. Complete Case Analysis: Receiver Operating Characteristic (ROC) curve comparisons and corresponding Area Under the ROC curves (AUC). [file 12877_2021_2444_MOESM2_ESM.docx]

**ADDITIONAL FILE 2 – SENSITIVITY ANALYSIS**

| Table S1. Complete case analysis: Baseline characteristics …...……………………………… | p. 2 |
| --- | --- |
| Table S2. Complete case analysis: Reproducibility properties of unchanged patients at three months follow-up measurement. ………………………………………………………………. | p. 3 |
| Table S3. Complete case analysis: Mean change scores and internal responsiveness for improved, unchanged, and deteriorated patients..……………………………………………. | p. 4 |
| Figure S1. Complete Case Analysis: Receiver Operating Characteristic (ROC) curve comparisons and corresponding Area Under the ROC curves (AUC)...…………………….. | p. 6 |

| **Table S1**. **Complete case analysis:** Baseline characteristics of patients who died (n=39) compared to patients with complete cases at both follow-up measurements (n=118) and patients who were loss to follow-up (n=56)^a^. | | | | | | |
| --- | --- | --- | --- | --- | --- | --- |
| **Baseline characteristics** | **Patients who died** | **Completers for both assessments** | **P value^b^** | **Loss to  follow-up** | **P value^c^** |  |
|  | **(n=39)** | **(n=118)** |  | **(n=56)** |  |  |
| **Age, median (IQR 25;75)** | 79 (75;86) | 75 (72;80) | <0.001 | 76 (71;81) | 0.008 |  |
| range (years) | 71 – 98 | 70 – 93 |  | 70 – 93 |  |  |
| **Sex, male** | 28 (72) | 82 (70) | 0.785 | 36 (64) | 0.443 |  |
| **Housing situation** |  |  |  |  |  |  |
| independent | 32 (82) | 114 (97) | 0.002 | 52 (93) | 0.105 |  |
| not independent | 7 (18) | 4 (3) |  | 4 (7) |  |  |
| **Education** |  |  |  |  |  |  |
| ≤ high school | 30 (77) | 85 (72) | 0.550 | 42 (75) | 0.830 |  |
| > high school | 9 (23) | 33 (28) |  | 14 (25) |  |  |
| **CCI, median (IQR 25;75)** | 4 (2; 5) | 2 (1; 4) | <0.001 | 2 (1; 3) | <0.001 |  |
| **Frailty Index** |  |  |  |  |  |  |
| median (IQR 25;75) | 0.31 (0.21; 0.48) | 0.14 (0.07; 0.26) | <0.001 | 0.17 (0.06; 0.33) | <0.001 |  |
| lowest possible score | 0 (0) | 0 (0) |  | 0 (0) |  |  |
| highest possible score | 0 (0) | 0 (0) |  | 0 (0) |  |  |
| *Missing* | 4 (10) | - |  | 6 (11) |  |  |
| **Frailty Phenotype** |  |  |  |  |  |  |
| median (IQR 25;75) | 2.00 (2.00; 3.00) | 0 (0; 2.00) | <0.001 | 1 (0, 2.00) | 0.002 |  |
| robust (0 criteria) | 4 (10) | 62 (53) | <0.001 | 21 (38) | 0.008 |  |
| prefrail (1 or 2 criteria) | 20 (51) | 41 (34) |  | 19 (34) |  |  |
| frail (≥3 criteria) | 13 (33) | 15 (13) |  | 11 (20) |  |  |
| lowest possible score | 4 (10) | 62 (53) |  | 21 (38) |  |  |
| highest possible score | 3 (8) | 4 (3) |  | 4 (7) |  |  |
| *Missing* | 2 (5) | - |  | 5 (9) |  |  |

Differences between groups were tested using Mann-Whitney U test in case of continuous variables, and Chi Square test in case of categorical variables. No differences were found between patients with complete cases at both follow-up measurements and patients lost to follow-up (all p values > 0.05).

1. All participants who withdrawn consent during the study have given their explicit consent that the data obtained so far could be used for scientific purposes.
2. Differences between patients who died and patients with complete cases at both follow-up measurements.
3. Differences between patients who died and patients lost to follow-up.

| **Table S2. Complete case analysis:** Reproducibility properties of unchanged patients at three months follow-up measurement. | | | | | | | |
| --- | --- | --- | --- | --- | --- | --- | --- |
| **Health anchor (n = 57)** | | | | | | | |
| **Instrument** | | | **ICC (95% CI)** | **SEM** | **SEM%^a^** | **SDC** | **SDC%^a^** |
|  | **FI** | | 0.83 (0.70; 0.91) | 0.04 | 4% | 0.12 | 12% |
|  | **FP** | | 0.62 (0.42; 0.76) | 0.59 | 12% | 1.64 | 33% |
| **Functioning anchor (n = 60)** | | | | | | | |
| **Instrument** | | | **ICC (95% CI)** | **SEM** | **SEM%^a^** | **SDC** | **SDC%^a^** |
|  | | **FI** | 0.88 (0.79; 0.93) | 0.04 | 4% | 0.12 | 12% |
|  | | **FP** | 0.63 (0.45; 0.76) | 0.57 | 11% | 1.59 | 32% |
| Notes: Intraclass correlation coefficient for agreement using a 2 way mixed effect model. ICC, Intraclass Correlation Coefficient, FI, Frailty Index; FP, Frailty Phenotype; SDC, smallest detectable change; SEM, standard error of measurement.   1. SEM% and SDC% are SEM and SDC expressed in percentages of the continuous score of the instrument. | | | | | | | |

| **Table S3. Complete case analysis:** Mean change scores and internal responsiveness for improved, unchanged, and deteriorated patients. | | | | | | | | | |
| --- | --- | --- | --- | --- | --- | --- | --- | --- | --- |
| **Health anchor** |  | **3 months post discharge** | **Spearman** | **SRM** |  | **12 months post discharge** | **Spearman** | **SRM** |  |
|  | **n** | **mean change score (SD)** | **Correlation** |  | **n** | **Mean change score (SD)** | **Correlation** |  |  |
| **Frailty Index** |  | | | | | | | |  |
| improved | 31 | -0.06 (0.10) | 0.32 | -0.60 | 34 | -0.06 (0.11) | 0.26 | -0.64 |  |
| unchanged | 57 | -0.02 (0.05) |  | -0.40 | 63 | -0.01 (0.08) |  | -0.13 |  |
| deteriorated | 30 | 0.02 (0.07) |  | 0.28 | 21 | 0.02 (0.10) |  | 0.22 |  |
| **Frailty Phenotype** |  | | | |  | | | |  |
| improved | 31 | -0.84 (1.24) | 0.33 | -0.68 | 34 | -0.82 (1.31) | 0.26 | -0.66 |  |
| unchanged | 57 | -0.02 (0.78) |  | -0.03 | 63 | -0.13 (0.87) |  | -0.15 |  |
| deteriorated | 30 | 0.13 (0.82) |  | 0.16 | 21 | 0.14 (1.15) |  | 0.12 |  |
| **Functioning anchor** | |  |  |  |  |  |  |  |  |
| **Frailty Index** |  | | | | | | | |  |
| improved | 24 | -0.07 (0.11) | 0.30 | -0.64 | 19 | -0.06 (0.12) | 0.17 | -0.50 |  |
| unchanged | 60 | -0.02 (0.06) |  | -0.33 | 68 | -0.01 (0.09) |  | -0.11 |  |
| deteriorated | 34 | 0.01 (0.07) |  | 0.14 | 31 | -0.01 (0.10) |  | 0.10 |  |
| **Frailty Phenotype** |  | | | |  | | | |  |
| improved | 24 | -0.89 (1.39) | 0.25 | -0.64 | 19 | -1.00 (1.49) | 0.23 | -0.67 |  |
| unchanged | 60 | -0.03 (0.75) |  | -0.04 | 68 | -0.19 (0.85) |  | -0.22 |  |
| deteriorated | 34 | 0.01 (0.85) |  | 0.01 | 31 | -0.03 (1.22) |  | -0.02 |  |

|  | 3 months post discharge | 12 months post discharge |
| --- | --- | --- |
|  | **Health anchor** | |
| improvement | 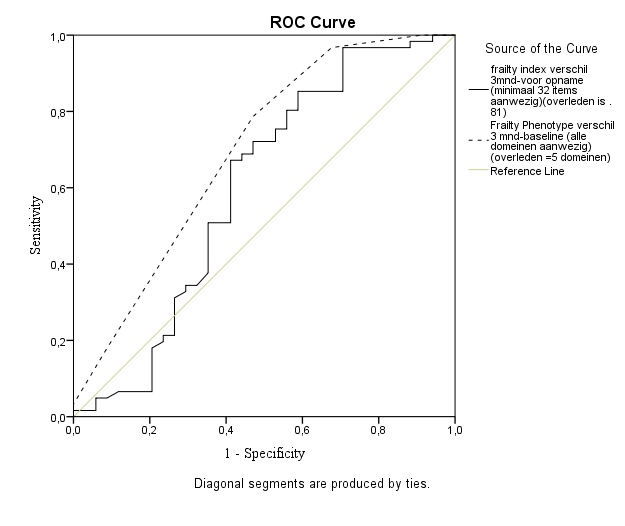  **AUC (95% CI)**  FI: 0.61 (0.48; 0.74)  FP: 0.70 (0.58; 0.82) | 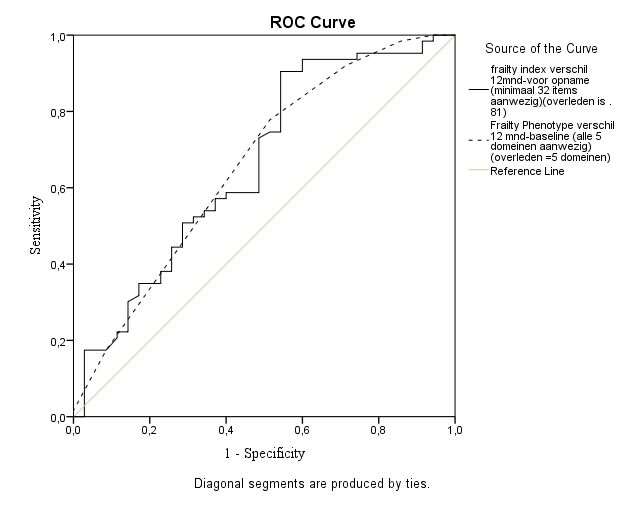  **AUC (95% CI)**  FI: 0.65 (0.53; 0.77)  FP: 0.65 (0.53; 0.77) |
| deterioration | 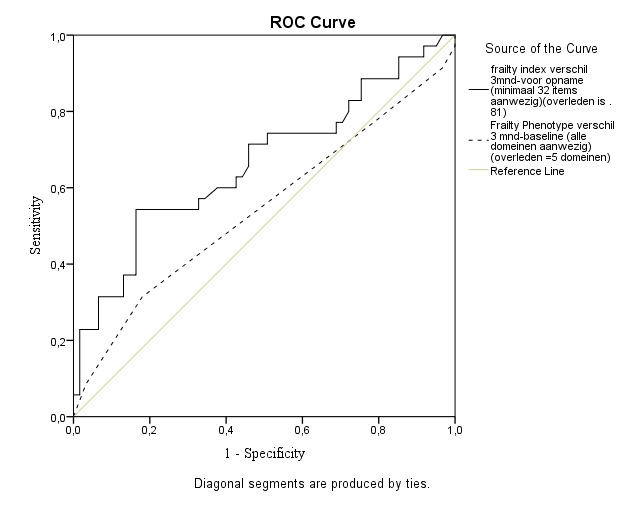  **AUC (95% CI)**  FI: 0.68 (0.55;0.81)  FP: 0.56 (0.43; 0.69) | 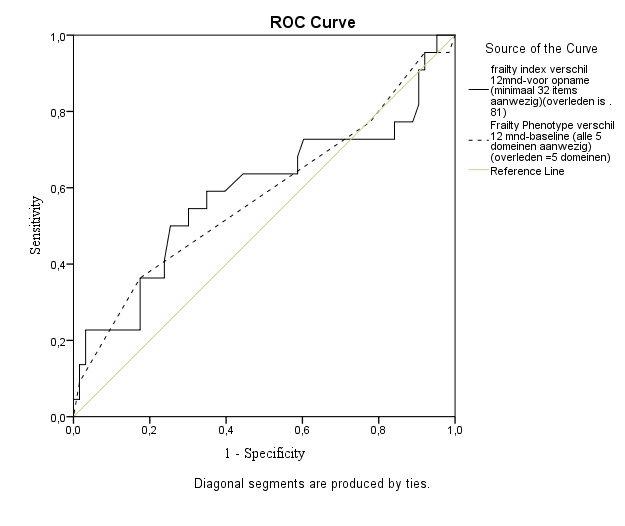  **AUC (95% CI)**  FI: 0.58 (0.42;0.74)  FP: 0.58 (0.43; 0.73) |

| Improvement | n=31 |  | n = 34 |
| --- | --- | --- | --- |
| Unchanged | n=57 |  | n = 63 |
| Deterioration | n=30 |  | n = 21 |

|  | **Functioning anchor** | |
| --- | --- | --- |
| improvement | 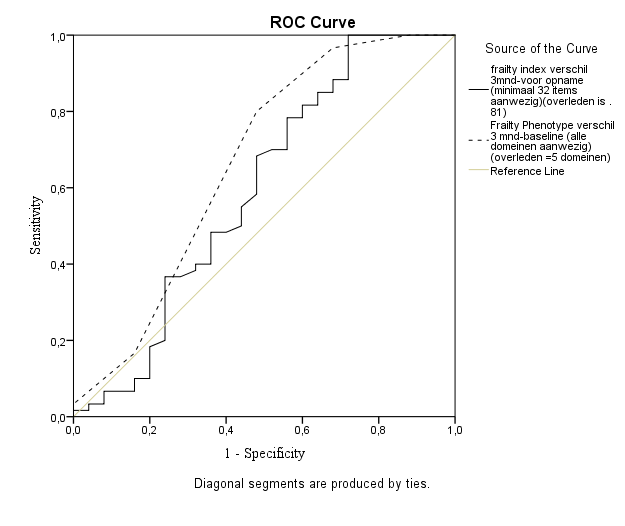  **AUC (95% CI)**  FI: 0.62 (0.47; 0.77)  FP: 0.67 (0.53; 0.82) | 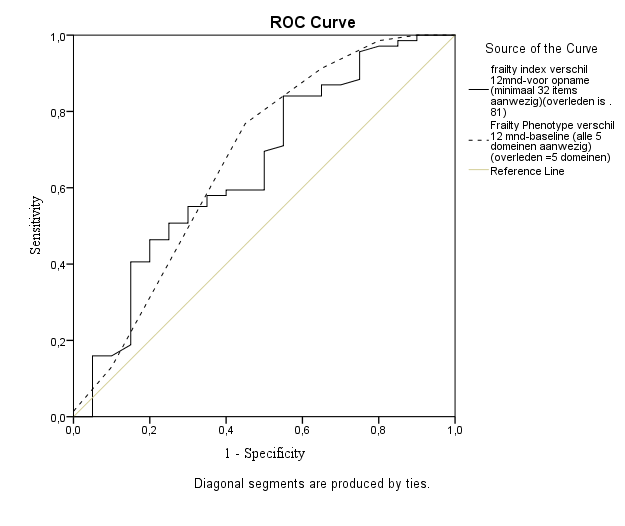  **AUC (95% CI)**  FI: 0.63 (0.49; 0.78)  FP: 0.66 (0.50; 0.81) |
| deterioration | 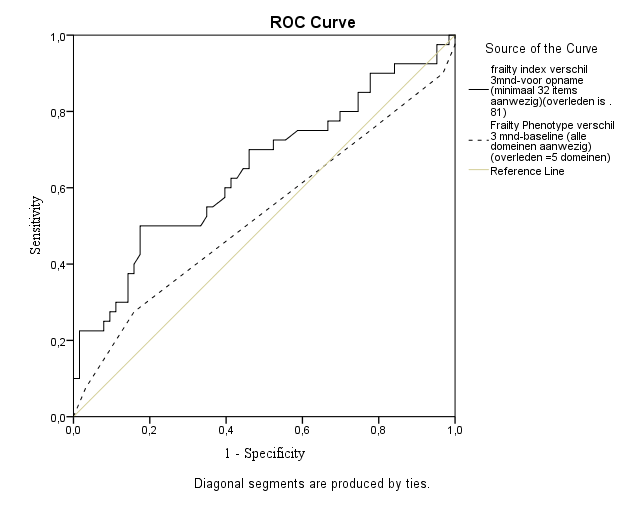  **AUC (95% CI)**  FI: 0.64 (0.52;0.76)  FP: 0.52 (0.40; 0.65) | 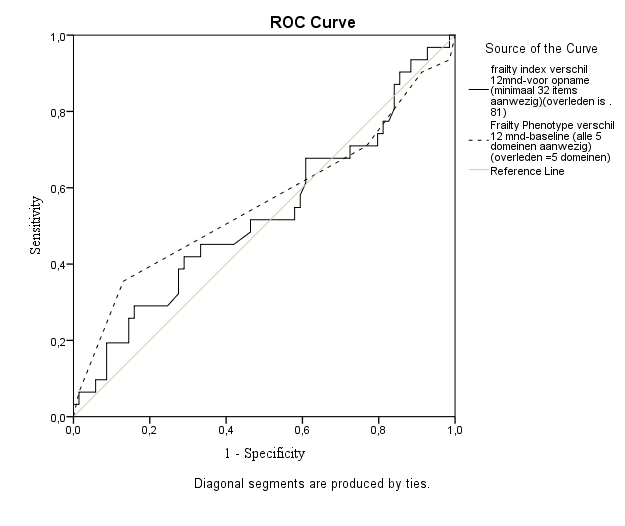  **AUC (95% CI)**  FI: 0.53 (0.40;0.66)  FP: 0.56 (0.43; 0.70) |

| Improvement | n=24 |  | n = 19 |
| --- | --- | --- | --- |
| Unchanged | n=60 |  | n = 68 |
| Deterioration | n=34 |  | n = 31 |

**Figure S1. Complete Case Analysis:** Receiver Operating Characteristic (ROC) curve comparisons and corresponding Area Under the ROC curves (AUC) between the Frailty Index (solid line) and the Frailty Phenotype (dashed line) for measuring improvement and deterioration in frailty status according to the health and functioning anchor questions after three (left) and twelve (right) months post discharge. The diagonal lines (gray) represent the reference lines of no-discrimination.
